# A Contribution Toward Understanding Trematode Diversity in Freshwater Snails of the Southwestern Kathmandu Valley, Nepal

**DOI:** 10.1155/jotm/1315939

**Published:** 2026-05-16

**Authors:** Minu Shilpakar, Rajendra Prasad Parajuli, Nishan Limbu, Mahendra Maharjan

**Affiliations:** ^1^ Central Department of Zoology, Tribhuvan University, Kirtipur, Kathmandu, Bagmati, Nepal, tribhuvan-university.edu.np; ^2^ Herbert Wertheim School of Public Health and Human Longevity Science, University of California San Diego (UCSD), San Diego, California, USA, ucsd.edu

**Keywords:** cercariae larvae, freshwater snails, intermediate hosts, prevalence, trematode infection

## Abstract

**Background:**

Freshwater snails serve as intermediate hosts for trematode parasites and play a key role in transmitting various parasitic diseases. Snail‐borne trematodiases pose important public and veterinary health risks in South Asia, where freshwater snails sustain transmission in agricultural and periurban waters. However, data from the Kathmandu Valley remain limited and fragmented across habitats and seasons.

**Objectives:**

This study aimed to determine the diversity and distribution of cercarial larvae in freshwater snail species from the southwestern part of the Kathmandu Valley, Nepal.

**Methods:**

From July to October 2023, a total of 1893 snail samples were collected from grazing swamps, ditches, paddy fields, and canals. The samples represented five genera within four families: *Lymnaea acuminata* and *Radix* spp. (Lymnaeidae), *Gabbia orcula* (Bithyniidae), *Physa acuta* (Physidae), and *Indoplanorbis exustus* (Planorbidae). Cercarial infections were examined using the light‐induced shedding method.

**Results:**

Four of the five snail species examined harbored trematode cercariae, with an overall prevalence of 3.01% (57/1893), while *Radix* spp. showed no infection. Nine cercarial morphotypes were identified: *Amphistome, Mutabile, Cercariaeum, Echinostome, Gymnocephalus, Cystophorous, Parapleurolophocerous, Xiphidiocercariae*, and *Brevifurcate apharyngate distome cercariae*. The infection prevalence varied significantly by month (Fisher’s exact test, *p* = 0.001), peaking in July (5.56%), August (4.95%), and by habitat (*p* = 0.001), with the highest prevalence in grazing swamps (4.91%). A significant positive correlation was observed between snail length and infection rate (*r* = 0.781, *p* < 0.001), with medium‐sized Lymnaea (18–26 mm) and Gabbia (10–13.9 mm) exhibiting the highest infection rates.

**Conclusions:**

Elevated infection occurred in July‐August and among larger swamp‐dwelling snails. The presence of diverse trematode cercariae, including zoonotically important types, underscores potential public and veterinary health risks and highlights the need for further studies on definitive hosts and control strategies. These patterns provide actionable guidance for risk mapping and prioritizing seasonal, habitat‐focused surveillance and control in the Kathmandu Valley.

## 1. Introduction

Freshwater snails act as intermediate hosts for different trematode parasites that belong to the superfamilies Fasciolidae, Schistosomatoidea, Paramphistomidae, Echinostomatoidea, Clinostomoidea, Diplostomoidea, and Pronocephaloidea [1, 2]. They play a vital role in the transmission of several snail‐borne parasitic diseases (SBPDs), such as fascioliasis, paramphistomiasis, and schistosomiasis, which can infect buffalo and other ruminants, as well as humans, causing significant economic losses [3].

The life cycle is complex and requires one definitive host and one or more intermediate hosts, progressing through intramolluscan stages including sporocysts and rediae, from which cercariae are produced. The cercarial stage does not live freely within snail tissues; rather, it develops inside sporocysts/rediae and is subsequently shed from the snail as a free‐swimming larva. After emergence, it either encysts as a metacercariae on aquatic vegetation or infects a second intermediate host, depending on the species [4, 5].

In Nepal, trematode parasitic infections are common health problems, particularly in domestic animals. These infections, such as fascioliasis, schistosomiasis, and paramphistomiasis, deteriorate the health conditions of the animals and indirectly affect the farmers’ economy. The spread of these parasitic diseases is locality specific, where a suitable intermediate host, snail distribution, is prevalent. Fascioliasis mainly occurs in cattle and domestic animals while they graze because they ingest plant matter grown in contaminated areas [6].

Snail‐borne trematode infections, therefore, represent an important ecological and socioeconomic interface linking aquatic environments, livestock production systems, and human health. In agrarian settings such as Nepal, where livestock farming contributes substantially to household income and food security, trematode infections reduce productivity through decreased weight gain, milk yield, and increased veterinary costs. Understanding site‐specific patterns of snail infection is, therefore, essential for risk assessment, targeted surveillance, and evidence‐based control strategies. Although cercarial infections have been reported from multiple districts in Nepal, existing evidence remains largely site‐specific and methodologically heterogeneous, with few studies simultaneously assessing season‐, habitat‐, and size‐related infection patterns within a unified framework for the Kathmandu Valley. To address this gap, we conducted a focused survey at four sites in the southwestern Kathmandu Valley selected for their pasture‐linked aquatic habitats (grazing swamps, canals, ditches, and paddy fields). Specifically, we aimed to (i) identify freshwater snail species present at these sites, (ii) determine the diversity of emergent trematode cercariae, and (iii) evaluate season‐, habitat‐, and size‐related patterns of infection to inform local risk mapping and guide targeted surveillance strategies.

## 2. Materials and Methods

### 2.1. Study Area

**FIGURE 1:**
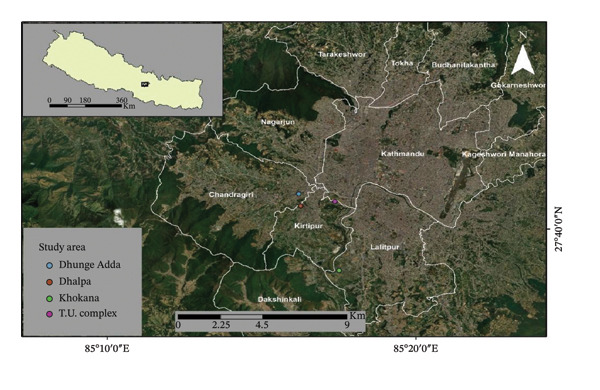
Map of the study area depicting tentative sampling points with different colors to identify.

The research was conducted on the outskirts of Kathmandu, which is situated at coordinates 27.71°N 85.32°E [7]. A preliminary survey was carried out between May and July 2023 to confirm the presence of freshwater snails in the study area before fieldwork was initiated. After confirmation, snail samples were collected from July to October 2023 from four distinct sites in the southwestern region of the Kathmandu Valley using an opportunistic sampling approach [8]. These sites were chosen on the basis of varying habitat features, including grazing swamps, canals, ditches, and paddy fields, to better understand the infection rate under diverse environmental conditions (Figure 1). The coordinates of all the sampling sites were recorded utilizing the global positioning system (GPS), specifically the Garmin eTrex 10 model. The specific coordinates were as follows: Khokana (27.64979, 85.28748), Dhalpa (27.68204, 85.27042), T.U. complex (27.68292, 85.29044), and Dhunge Adda (27.68729, 85.27355). Most residents in this area are engaged in agriculture and raise livestock such as cows, buffaloes, and goats. All four sites contained a mosaic of habitat types (grazing swamps, canals, ditches, and paddy fields); thus, habitats were sampled within sites (nested design) rather than representing a single habitat per site.

### 2.2. Methods

#### 2.2.1. Sample Collection and Preservation

Live snails were gathered from various sites using forceps and manual picking techniques while wearing protective gloves. The collection at each site lasted approximately 30 min. The snails were subsequently placed into plastic containers filled with water and labeled appropriately. The containers were transferred to the Central Department of Zoology, Kirtipur Laboratory, for additional analysis and identification. Habitats were predefined in the field as grazing swamps (seasonally inundated, vegetation‐rich areas used by livestock), canals (engineered channels with slow flow), ditches (shallow roadside or field margins with stagnant/slow water), and paddy fields (flooded rice plots). One site could contain multiple habitat types, and sampling, therefore occurred within habitats nested inside sites (partially crossed).

### 2.3. Morphological Identification of Freshwater Snails

The collected snails were cleaned with distilled water to remove mud, leaf litter, algae, and debris and then identified to the species level using established malacological keys based on diagnostic shell morphology, including shell shape, size, color, aperture form, number of whorls, and spire characteristics [9, 10].

### 2.4. Extraction and Morphological Identification of Cercariae

**FIGURE 2:**
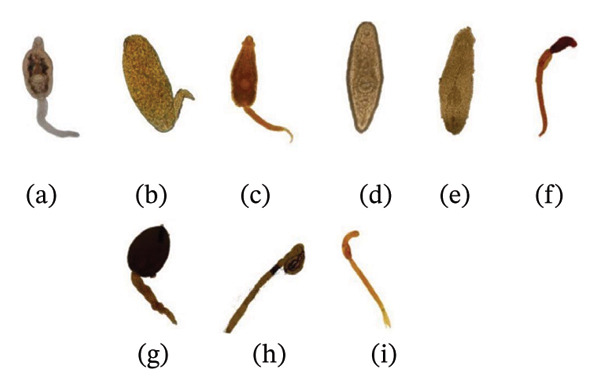
Microscopy images of different types of trematode larvae that emerged from freshwater snails. (a–i) Cercariae, (a) *Gymnocephalus* sp., (b) *Xiphidocercariae* sp., (c) *Echinostome* sp., (d) *Cercariaeum* sp., (e) *Mutabile* sp., (f) *Parapleurolophocercous*, (g) *Amphistome s*, (h) *Cystophorous* sp., and (i) *Brevifurcate apharyngate distome* sp.

Larval trematode cercariae were examined using the shedding method [11]. Snails were kept individually in clean cavity blocks with distilled water and exposed to sunlight for 1 to 2 h. After exposure, the water samples were examined on a watch glass under a microscope for cercariae. Snails that did not shed cercariae were retested on subsequent days. Emergent cercariae were stained with one drop of iodine solution and allowed to stand for approximately 1 min, and then 1 to 2 drops of the sample were placed on a clean slide with a cover slip. Slides were examined under a stereomicroscope (10x) and photographed using an Olympus CX43 microscope, and cercarial morphotypes were identified (Figure 2) based on published morphological criteria using standard keys, calibrated eyepiece micrometry, and photodocumentation by two independent readers, with discrepancies resolved by consensus [11–13].

### 2.5. Data Analysis

The raw data were entered into Microsoft Excel for preliminary processing and descriptive analysis. Prevalence was calculated as the percentage of infected snails among the total snails examined. Snail species were grouped into size classes to assess sizewise infection patterns. Differences in infection rates across months and habitats were analyzed via chi‐square or Fisher’s exact tests, and correlations between snail length and infection rate were evaluated using Spearman’s rank correlation in R (v4.2.1). All statistical tests were two‐sided, and statistical significance was defined a priori at *p* < 0.05.

## 3. Results

### 3.1. Identification of Freshwater Snails

A total of 1893 freshwater snails were collected from the four different study areas. The collected snails were distinguished into four families and five genera: *Lymnaea acuminata* and *Radix* spp. (Lymnaeidae), *Gabbia orcula* (Bithyniidae), *Physa acuta* (Physidae), and *Indoplanorbis exustus* (Planorbidae). Among the 1893 snail species, 886 *Lymnaea acuminata*, 293 *Gabbia orcula*, 692 *Physa acuta*, 20 *Indoplanorbis exustus*, and two *Radix* spp. were examined for the presence of cercariae.

The identified snail species included *Lymnaea acuminata* (Figure 3(a)), characterized by a large dextral shell with a short spire; *Radix* spp. (Figure 3(b)), showing a dextral shell with a short, blunt spire, and expanded outer lip; *Physa acuta* (Figure 3(c)), distinguished by a sinistral (left‐coiling) shell with a pointed spire; *Indoplanorbis exustus* (Figure 3(d)), exhibiting a discoid, planispiral shell typical of *Planorbidae*; and *Gabbia orcula* (Figure 3(e)), identified by a small globular shell with an operculum and conical whorls.

FIGURE 3Shell morphology of snail species of different families; (a) *Lymnaea acuminata*; (b) *Radix* spp.; (c) *Physa acuta*; (d) *Indoplanorbis exustus*; (e) *Gabbia orcula*.

**Figure:**
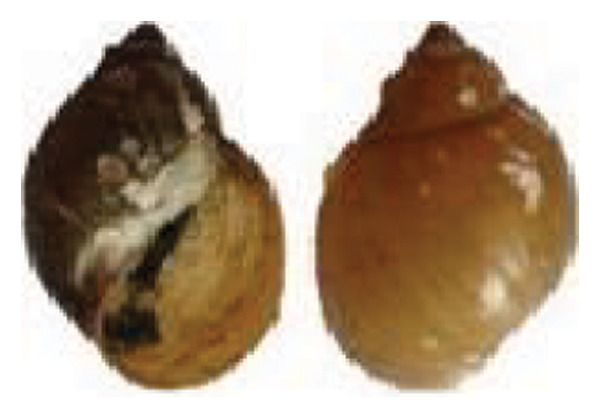
(a)

**Figure:**
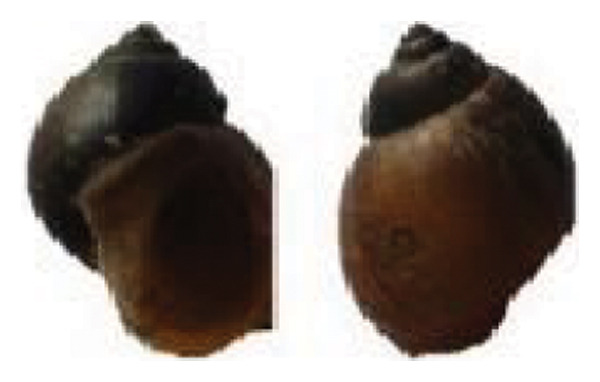
(b)

**Figure:**
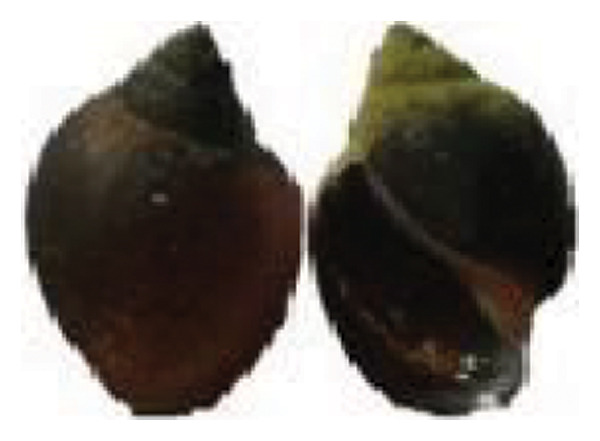
(c)

**Figure:**
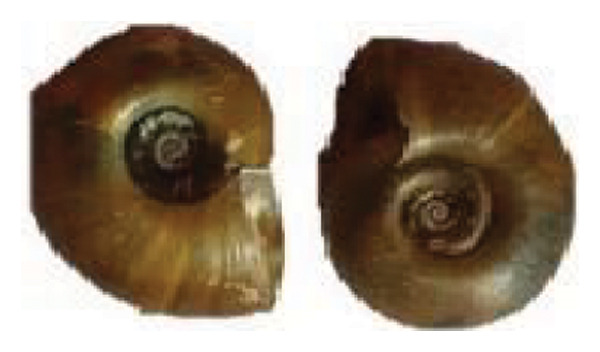
(d)

**Figure:**
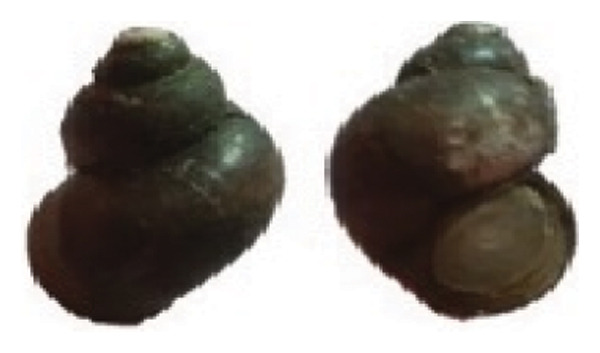
(e)

### 3.2. Identification of Trematode Larvae

Cercariae were identified on the basis of key morphological traits, including the position and number of suckers, arrangement of internal organs, and tail structure (Table 1). Because some size classes contained few individuals, certain prevalence values (e.g., 66.67%) reflect small denominators and should be interpreted with caution. Nine morphologically distinct types (A–I) were recorded: *Gymnocephalus, Xiphidocercariae, Echinostome, Cercariaeum, Mutabile, Parapleurolophocercous, Amphistome, Cystophorous*, and *Brevifurcate apharyngate distome* (BAD) (Figure 2).

**TABLE 1:** Lengthwise infection prevalence of snail species (with sample size per size class).

Snail species	Size (mm)					
	2.0–5.9	6.0–9.9	10.0–13.9	14.0–17.9	18.0–21.9	22.0–26.0
*Lymnaea*	—	—	0.99 (6/606)	8.33 (9/108)	66.67 (2/3)	66.67 (2/3)
*Gabbia*	—	10.25 (28/273)	66.67 (2/3)	—	—	—
*Physa*	4.76 (1/21)	0.36 (2/598)	1.37 (1/73)	—	—	—
*Indoplanorbis*	—	—	12.5 (1/8)	33.33 (3/9)	—	—

*Gymnocephalus* cercariae possessed a circular anterior oral sucker without a stylet or collar, a midventral ventral sucker, numerous cystogenous glands, and an unforked tail (head/tail = 360.74 ± 24.67 μm/499.07 ± 77.12 μm). The *Xiphidocercariae* were small, flat, and oval with a short slender tail, an anterior oral sucker, and a smaller midbody ventral sucker (322.35 ± 51.70 μm/94.38 ± 7.76 μm). *Echinostome* cercariae presented an unforked tail with a thick tip, an oral sucker encircled by a weak spiny collar, and no eyespots (215.10 ± 42.82 μm/232.63 ± 59.35 μm). *Cercariaeum* types lack a tail, with a tapered body, subterminal oral sucker, medial ventral sucker, and bifurcated esophagus (415.91 ± 57.85 μm). *The mutabile* forms were fusiform, with subterminal oral and midbody ventral suckers and bifurcated intestinal caeca (580.70 ± 34.27 μm). *Parapleurolophocercous* cercariae had a long unforked tail bearing lateral and dorsoventral fin folds, vestigial or absent ventral suckers, and eyespots (111.12 ± 72.47 μm/356.98 ± 225.21 μm). *The amphistome* cercariae were characterized by an unforked tail, a large posterior ventral sucker, and numerous cystogenous glands (145.28 ± 64.01 μm/187.67 ± 82.90 μm). The carnivorous type possesses a large tail with an anterior chamber into which the body can be retracted (1106.03 ± 74.81 μm). *The* BAD cercariae feature a bifurcate tail, oral and ventral suckers, and the absence of a pharynx (92.01 ± 26.52 μm/323.61 ± 25.46 μm).

### 3.3. Trematode Infection in Freshwater Snails

**FIGURE 4:**
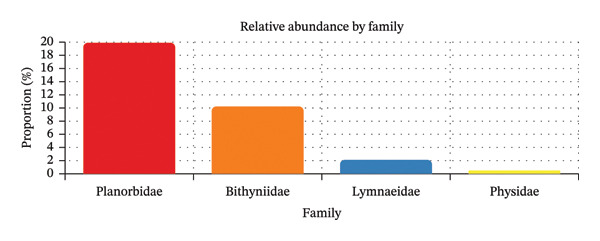
Prevalence of trematode infection in each snail family.

The overall prevalence of trematode infection among the collected freshwater snails was 3.01% (57/1893). Among the infected families, Planorbidae (*Indoplanorbis exustus*) presented the highest susceptibility to trematode infection, with a prevalence rate of 20%, followed by Bithyniidae (10.24%), Lymnaeidae (2.14%), and Physidae (0.58%) (Figure 4). Snails of the family Lymnaeidae (*Radix* spp.) showed no detectable infection, but no inference is warranted given the small sample.

#### 3.3.1. Monthwise Trematode Infection

**FIGURE 5:**
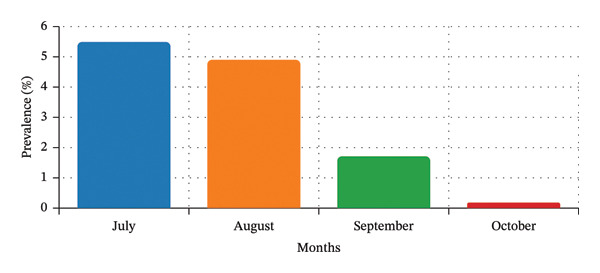
Monthwise trematode infection in freshwater snails.

The prevalence of trematode infection varied significantly across months (*p* = 0.001, Fisher’s exact test). The infection rates peaked in July (5.56%) and August (4.95%) and then declined in September (1.72%) and October (0.19%) (Figure 5). These results indicate a pronounced seasonal pattern, with markedly greater cercarial emergence during the early monsoon months.

#### 3.3.2. Habitatwise Trematode Infection

**FIGURE 6:**
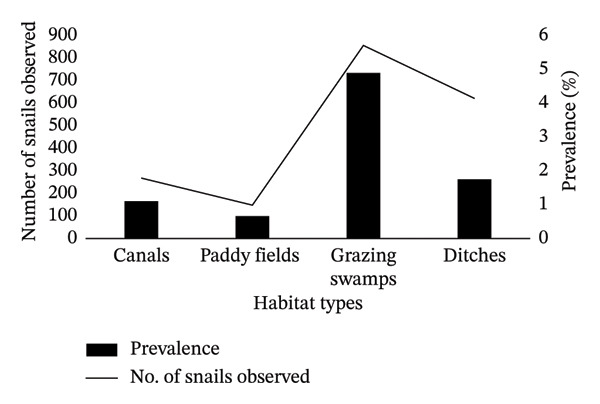
Habitatwise trematode infection: prevalence (%) and number examined (n).

Significant variation in infection prevalence was also observed across habitat types (*p* = 0.001). The highest infection rate occurred in snails collected from grazing swamps (4.91%), followed by those collected from ditches (1.77%), canals (1.12%), and paddy fields (0.68%) (Figure 6). This pattern suggests that stagnant and vegetation‐rich environments favor cercarial transmission and snail infection. To aid interpretation, we present habitat‐specific prevalence and the corresponding number examined (*n*) on a secondary axis in Figure 6. Note that habitat types were sampled within sites (i.e., sites could include multiple habitats), so prevalence is reported by habitat category with corresponding denominators. Counts (*n*) are shown for context and should not be interpreted as prevalence.

#### 3.3.3. Correlation Between Snail Length and Infection Rate

A strong positive correlation was observed between snail length and infection rate (*r* = 0.781, *p* < 0.001). Among *Lymnaea* species, medium‐sized individuals (18–26 mm) presented the highest infection rate (66.67%). Similarly, *Gabbia* snails measuring 10–13.9 mm presented the greatest degree of infection (66.67%), whereas *Physa* snails of the same size range presented a lower prevalence (1.37%). *For Indoplanobia*, the highest infection rate (33.33%) occurred in snails measuring 14–17.9 mm.

## 4. Discussion

Freshwater snails act as intermediate hosts for numerous trematode parasites of public and veterinary health significance. In Nepal, these snails are widely distributed across diverse aquatic habitats, from the lowland wetlands of the Terai to high‐altitude Himalayan lakes. Their habitat preferences vary considerably; some species favor slow‐flowing rivers and rocky substrates, whereas others thrive in stagnant or slow‐moving waters such as ponds, ditches, and paddy fields. This ecological diversity provides suitable conditions for maintaining trematode life cycles and sustaining transmission in different environmental settings. Although only 139 mollusk species have been officially documented in Nepal, the continual discovery of new species each year, coupled with high levels of endemism (94.6% among terrestrial and 47.8% among aquatic mollusks), highlights the country’s remarkable and unique biodiversity [14]. Dey and Mitra [15] recorded 92 freshwater mollusk species, 44 of which were endemic to the Himalayan region. Several studies have examined the status of trematode infections in freshwater snails across different districts of Nepal; however, information remains fragmented and region specific. The present study identified five freshwater snail species, *Lymnaea acuminata, Gabbia orcula, Physa acuta, Indoplanorbis exustus*, and *Radix* spp., belonging to four families. Among these, *L. acuminata*, *G. orcula*, and *P. acuta* were cosmopolitan, occurring at all four study sites. Previous research has reported that approximately nine snail species from six families are infected by the trematode cercariae in Nepal [9], further supporting the country’s diverse potential for hosting intermediate stages of trematodes.

This study demonstrated that the four‐snail species act as intermediate hosts of trematodes, namely, *Lymnaea acuminata, Gabbia orcula, Physa acuta,* and *Indoplanorbis exustus*. The diversity of cercariae found in infected snail species comprises Gymnocephalus, Cercariaeum, Xiphidocercariae, Echinostome, Mutabile, Parapleurolophocercous Amphistome, Cystophorous, and BAD, with these cercarial types belonging to the adult stages of Fasciolidae [11, 16], Cyclocoelidae [11, 17], Lecithodendriidae [11, 16–18], Echinostomatidae [11, 12, 17, 19, 20], Monorchioidea [5, 11, 13, 16, 19, 21, 22], Paramphistomoidea [23–25], Hemiuridae [11, 13, 23, 26, 27], and Schistosomatidae [9, 11] trematodes, respectively. These families have been reported as trematodes of vertebrates, especially birds, reptiles, fish, and mammals.

The release of human *Schistosome* cercariae (BAD) from *Lymnaea acuminata* could be linked to the transmission of *Schistosoma* infection in the study areas. However, only a few reports related to the transmission of *Schistosoma* infection in humans have been reported in Nepal until now; hence, the presence of *schistosome* cercariae represents a risk of potentially active schistosomiasis foci. A case related to urinary schistosomiasis in humans has been reported in a 34‐year‐old male from Siraha District, and a 23‐year‐old female visited CIWEC Hospital and Travel Medicine Center, Kathmandu [28, 29]. This blood fluke causes human cercarial dermatitis and directly impacts cattle, birds, reptiles, fish, and mammals [11]. Because of their grazing habits and wallowing nature, cattle are likely to be more exposed to Schistosoma infections. Similarly, gymnocephalus and amphistome cercariae are *Fasciola* spp. and *Paramphistomum* spp. These two common parasites can infect cattle and sheep, impacting their health and, in severe cases, leading to their death, which poses a significant threat to the livestock industry [30]. In the Republic of Korea, the most frequently reported foodborne intestinal trematodes from the Echinostomatidae and Heterophyidae families are transmitted to humans through the consumption of raw or undercooked foods such as fish and oysters. The primary reservoir hosts for these trematodes include dogs, cats, rats, ducks, and oystercatchers [31].

In this study, freshwater snails from different areas of the Kathmandu Valley acted as intermediate hosts for various zoonotic trematodes, with an overall prevalence of 3.01%. The prevalence of cercarial infection showed a distinct seasonal pattern, peaking in July (5.56%) and reaching its lowest level in October (0.59%). A similar pattern was reported in an earlier study [32], which reported the highest infection rate in July (40.11%), supporting the role of seasonal factors in trematode transmission. The observed differences in infection rates between studies may be attributed to variations in snail abundance, species diversity, local ecological conditions, and sampling intensity. Further studies across different ecological and climatic zones are needed to confirm and better understand these seasonal dynamics.

The prevalence of cercarial infection was highest in grazing swamps (4.91%), likely due to open grazing activities, the presence of migratory and resident birds, and frequent human and livestock interactions. This prevalence was higher than that reported in earlier studies [9] for grazing swamps (3.2%) but lower than that reported for canals (12%) and paddy fields (10%) [26]. In contrast, the paddy fields in the present study presented the lowest infection rate (0.68%), which may be attributed to reduced human activity and limited livestock presence. These findings indicate that ecological and biological factors, such as water movement, organic matter availability, and animal density, shape cercarial transmission, with the diverse aquatic habitats of the southwestern Kathmandu Valley facilitating cercarial development and sustained snail–parasite interactions.

The present study revealed a positive correlation between snail length and trematode infection, indicating that larger snails were more frequently infected. The occurrence and intensity of digenetic trematode infections in snail intermediate hosts are influenced by multiple factors, including snail size, light exposure, temperature fluctuations, and water depth [33, 34]. Similar findings were reported in an earlier study [35], which reported that infection rates increased with snail length in *Nerita* species. These findings suggest that, owing to their greater surface area and longer lifespan, larger snails provide more favorable conditions for parasite establishment and development. However, further studies involving controlled environmental parameters are needed to confirm this relationship.

Because the overall prevalence was low (3.01%) and several habitat‐by‐month strata were sparse, a unified multivariable model (e.g., including site, habitat, month, interactions, and snail size) was underpowered; therefore, we report prevalence and limited hypothesis tests. Future work should use a stratified, adequately powered design to enable comprehensive modeling. In addition, as sampling was opportunistic and confined to four nearby sites, findings may not fully represent all habitat variability within the Kathmandu Valley; future surveys should employ stratified random sampling with replicated transects or quadrats to improve representativeness and control for habitat‐level detectability. This morphotype‐based approach may not distinguish cryptic taxa or closely similar morphotypes; future work should incorporate molecular barcoding to confirm lineage identity and refine diversity estimates. The very small number of Radix spp. sampled (*n* = 2) limited inferential comparisons; future work will target additional Radix sampling to ensure adequate power. Although no infections were detected in *Radix* spp., the small sample size limits conclusive interpretation. Although zero‐inflated and hurdle models are well suited to sparse ecological datasets, the very low infection prevalence and sparse cell counts across habitat‐by‐month strata in this study precluded their robust application.

## 5. Conclusions

This study identified five freshwater snail species in southwestern Kathmandu Valley, four of which (*Lymnaea acuminata*, *Gabbia orcula*, *Physa acuta*, and *Indoplanorbis exustus*) harbored nine trematode cercarial morphotypes, including potentially zoonotic taxa. The overall infection prevalence was 3.01%, peaking in July‐August and peaking in grazing swamp habitats. A positive correlation between snail length and infection rate suggests that larger snails are more vulnerable to trematode colonization. These results emphasize the ecological and epidemiological importance of snail–parasite systems in the region. We recommend integrated snail surveillance, habitat management (especially swamps), and molecular characterization of cercariae across ecological zones to better assess and mitigate trematode transmission risk.

## Author Contributions

Minu Shilpakar: conducted the fieldwork, sample and data collection, and lab investigation and drafted the manuscript. Rajendra Prasad Parajuli: study design, data analysis, interpretation, and revision Nishan Limbu: study design, parasite identification, and manuscript revision. Mahendra Maharjan: overall supervision and manuscript revision.

## Funding

The authors did not receive support from any organization for the submitted work.

## Disclosure

The research paper is a part of the MSc thesis of the first author Minu Shilpakar.

## Ethics Statement

No approval from research ethics committees was required to accomplish the goals of this study because the experimental work was conducted with an unregulated invertebrate species. All specimens were collected under strict hygienic conditions, and the procedures for handling and sampling adhered to relevant institutional and national ethical guidelines for animal research.

## Conflicts of Interest

The authors declare no conflicts of interest.

## Data Availability

The data that support the findings of this study are available from the authors upon reasonable request. The authors are responsible for the correctness of the statements provided in the manuscript.
